# Structural insights of AKT and its activation mechanism for drug development

**DOI:** 10.1007/s11030-025-11132-7

**Published:** 2025-02-26

**Authors:** B. Harish Kumar, Shama Prasada Kabekkodu, K. Sreedhara Ranganath Pai

**Affiliations:** 1 Department of Pharmacology, Manipal College of Pharmaceutical Sciences, Manipal Academy of Higher Education, Manipal, Karnataka 576104 India; 2 Department of Applied Biology, CSIR-Indian Institute of Chemical Technology (IICT), Hyderabad, 500007 India; 3 Department of Cell and Molecular Biology, Manipal School of Life Sciences, Manipal Academy of Higher Education, Manipal, Karnataka 576104 India

**Keywords:** PH domain, AKT active conformation, Hydrophobic motif, AlphaFold structure, Phosphorylation, AKT1 activation, AlphaFold’s limitations

## Abstract

AKT1, a serine/threonine kinase, is pivotal in signaling and regulating cell survival, proliferation, and metabolism. This review focuses on the structural insights and the essential features required for its active conformation. AKT belongs to the AGC kinase group and has three isoforms: AKT1, AKT2, and AKT3. AKT has three functional regions: PH domain, kinase domain, and hydrophobic motif. AKT1 activation involves intricate conformational changes, including transitions in the αC-in, DFG-in, G-loop, activation loop, and PH domain out, S-spine and R-spine formation, as well as phosphorylation at Thr 308 and Ser 473, which enable AKT1 to adopt active conformation. The analysis highlights the limitations of the AlphaFold-predicted AKT1 structure, which lacks key elements of the active state, including ATP, magnesium ion coordination, phosphatidylinositol-(1,3,4,5)-tetraphosphate, substrate peptide, and phosphorylation at Thr 308 and Ser 473. This study underscores the necessity of these features for stabilizing the kinase domain and facilitating efficient substrate phosphorylation. By consolidating structural insights and activation mechanisms, this review aims to inform the development of computational models and targeted therapeutics for AKT1 activators in diseases such as hepatic ischemia–reperfusion injury, cerebral ischemia, acute hepatic failure, subarachnoid hemorrhage, and alzheimer’s disease.

## Introduction

**Fig. 1:**
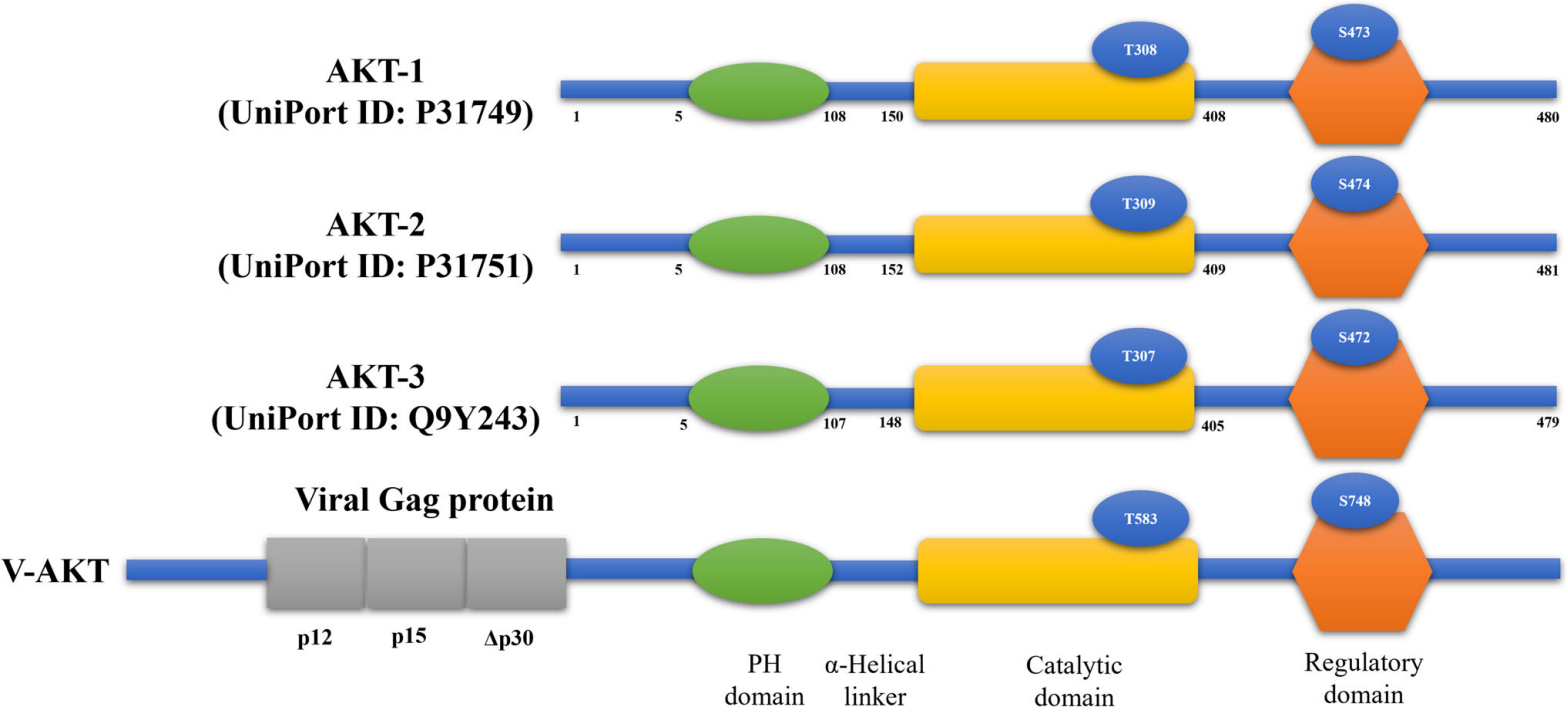
Structure of different AKT isoform in comparison with V-AKT

While doing virological research on tumor cell lines derived from spontaneous thymomas of the high-leukemia AKR mouse strain in 1977, Stephen Staal discovered the AKT8 murine retrovirus [1]. Later, in 1987, Stephen Staal used a modified, nonproducer cell line to molecularly clone AKT8 provirus. Both nonviral cell and viral-related sequences were included in the virus genome; the nonviral sequence was named v-AKT. In the same investigation, two human homologs of the v-AKT oncogene of the AKT8 provirus, AKT1, and AKT2, were discovered (Fig. 1) [2]. By 1988, Staal had localized the AKT1 gene to human chromosome 14 at band q32, near the heavy-chain immunoglobulin locus [3]. In 1991, three independent research groups cloned and characterized the cellular homologue of v-AKT, protein kinase (57 kDa) that phosphorylates serine and threonine residues. Bellacosa and Tsichlis cloned this kinase using cDNA hybridization with v-AKT and termed it c-AKT [4]. The Hemmings group employed degenerate PCR targeting sequences encoding catalytic domains of protein kinases and identified the kinase, naming it related to A and C-kinase (RAC) [5]. Meanwhile, Woodgett and Coffer used library screening to isolate a protein kinase, which they called protein kinase B (PKB) due to its resemblance to (protein kinase A) PKA and PKC [6]. In 1995, Richard Roth and colleagues demonstrated that AKT activation occurs in response to insulin stimulation, sparking significant interest in the regulatory mechanisms and functional roles of this kinase [7].

The AKT kinase family is recognized for its significant structural resemblance to PKA and protein kinase C (PKC). This family includes three isoforms AKT1 (PKBα), AKT2 (PKBβ), and AKT3 (PKBγ), which are highly conserved in mammalian genomes. These isoforms are encoded by distinct genes located on chromosomes 14q32 (12F1-2), 19q13 (7B1), and 1q44 (1H4-6), respectively [8, 9]. AKT1 is ubiquitously expressed across various tissues and plays a critical role in promoting cell growth and survival. In contrast, AKT2 is predominantly found in muscle tissue and adipocytes, where it facilitates insulin-regulated glucose homeostasis. AKT3 exhibits a more limited expression profile, being primarily localized to the brain and testes. In the brain, AKT2 is mostly expressed in astrocytes but not in hippocampus neurons, whereas AKT1 and AKT3 are dispersed throughout the somatic layers of the hippocampus [10].

Hakryul Jo et al. [11] in 2012 identified the first known AKT activators by screening chemical compounds from databases like ChemBridge, ChemDiv, Maybridge, and ChemDiv, using high-throughput cell-based assays. This screening led to the discovery of SC79, an ethyl 2-amino-6-chloro-4-(1-cyano-2-ethoxy-2-oxoethyl)-4H-chromene-3-carboxylate compound. SC79 is relatively unstable in water, capable of crossing the blood–brain barrier (BBB), and has been found to be non-toxic in both *in-vitro* and *in-vivo* models. Typically, AKT activation is initiated when its pleckstrin homology (PH) domain binds to phosphatidylinositol-3,4,5-trisphosphate (PIP₃) at the cell membrane, exposing phosphorylation sites for PDK1. However, SC79 directly binds to the PH domain of AKT in the cytosol, inducing a conformational change that facilitates its activation by PDK1, independent of membrane localization. Several researchers have explored the therapeutic potential of SC79 in many diseases such as hepatic ischemia–reperfusion injury [12], cerebral ischemia [13, 14], acute hepatic failure [15], subarachnoid hemorrhage [16], and Alzheimer’s disease [17].

Another research group while studying the mechanisms of phytochemicals beneficial for diabetes identified additional AKT-activating compounds, including chlorogenic acid [18], puerarin [19], quercetin-3-glucuronide [20], baicalin [21], kaempferol-3-glucuronide [20], ferulic-4-*O*-glucuronide [22], and Caffeic-4-*O*-glucuronide [22]. Despite these advancements, high-throughput virtual screening methodologies have not been widely applied, primarily due to the lack of a crystal structure of full-domain AKT in its active state. This review seeks to provide a detailed overview of the structure and activation mechanism of AKT while addressing structural limitations in AlphaFold’s models. By analyzing available structural data and highlighting critical features required for AKT’s active conformation, we aim to pave the way for developing enhanced computational models for drug discovery targeting the AKT pathway.

## AKT pathway

The AKT signaling pathway is a critical signal transduction mechanism that promotes cell survival and growth in response to extracellular signals via receptor tyrosine kinases (RTKs) or G-protein-coupled receptors (GPCRs). This pathway involves two key enzymes: phosphatidylinositol-3-kinase (PI3K) and AKT. Upon stimulation by growth factors, a cell surface receptor activates, leading to PI3K phosphorylation. Class I PI3K phosphorylates the 3′ hydroxyl group of the inositol head of phosphoinositides, generating lipid second messengers such as phosphatidylinositol-3,4,5-trisphosphate (PIP3) and phosphatidylinositol-3,4-bisphosphate (PIP2). PIP3 then recruits downstream effectors like AKT and 3-phosphoinositide-dependent kinase 1 (PDK1) via their PH domains [23].

In its autoinhibited state, AKT adopts a “PH-in” conformation due to intramolecular interactions between its PH and kinase domains. Upon PIP3 binding, AKT shifts to a “PH-out” conformation, exposing Thr 308/309/305 in the activation loop of AKT1/2/3, respectively, allowing phosphorylation by PDK1. This active, phosphorylated “PH-out” form can dissociate from the membrane to phosphorylate downstream substrates. Full activation of AKT requires additional phosphorylation at Ser 473/474/472 in the hydrophobic motif by mechanistic target of rapamycin complex 2 (mTORC2), which enhances Thr 308 phosphorylation stability and maximizes AKT activity. Without Ser 473 phosphorylation, AKT retains some activity, but it is significantly reduced [23].

The AKT pathway serves as a central signaling axis, and its dysregulation has been associated with various conditions, including developmental and overgrowth syndromes, cancer, cardiovascular diseases, insulin resistance, type 2 diabetes, inflammatory and autoimmune disorders, as well as neurological diseases such as alzheimer’s, parkinson’s, and stroke. A critical role of the AKT pathway is its ability to enhance cell survival by suppressing apoptosis. AKT achieves this by phosphorylating and deactivating pro-apoptotic proteins, such as Bad and caspase-9, thereby inhibiting the initiation of the apoptotic cascade. Additionally, AKT promotes cell survival by upregulating the expression of anti-apoptotic proteins like Bcl-2. Given its pivotal role in regulating the PI3K/AKT pathway, AKT represents a promising therapeutic target for a range of diseases.

## AKT structure

**Fig. 2:**
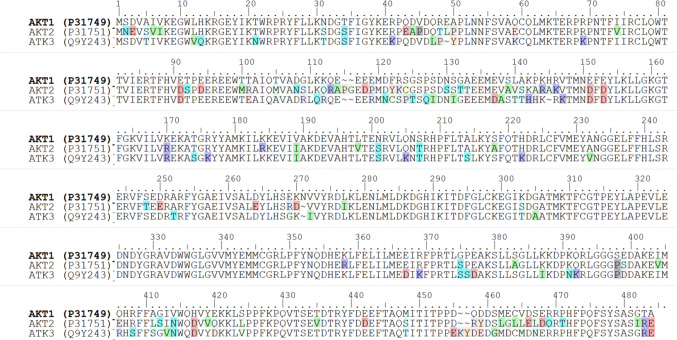
Sequence alignment of AKT isoforms, emphasizing conserved and divergent regions across isoforms

The AKT kinase protein belongs to the AGC kinase family. The structure of AKT is complex and can be described in terms of its domains and functional regions, which are essential for its activity and regulation. The AKT is a homolog of the v-AKT oncogene of the AKT8 provirus without the viral gag protein. The sequence of AKT1 is identical to AKT2 and AKT3 showing 81 and 82% amino acid identity with AKT1, respectively. AKT has three domains: the PH domain, kinase, and C-terminal tail (Fig. 2 and Table 1). The PH domain is a structurally well-characterized module of approximately 120 amino acids which is involved in the recruitment of AKT to the plasma membrane. The catalytic (kinase) domain is responsible for the enzymatic activity of AKT. The regulatory C-terminal tail (C-tail) is involved in the regulation of AKT’s activity.

**Table 1:** Percentage identity of various regions among AKT isoforms, including PH domain, linker, kinase domain, and terminal region

% Identity	Full	AKT1 PH domain (5–108)	AKT1 Linker region (109–149)	AKT1 Kinase domain (150–408)	AKT1 Terminal (409–480)
AKT1/AKT2	81	81	49	91	67
AKT1/AKT3	82	85	44	88	76
AKT2/ATK3	76	77	30	88	66

## PH domain

**Fig. 3:**
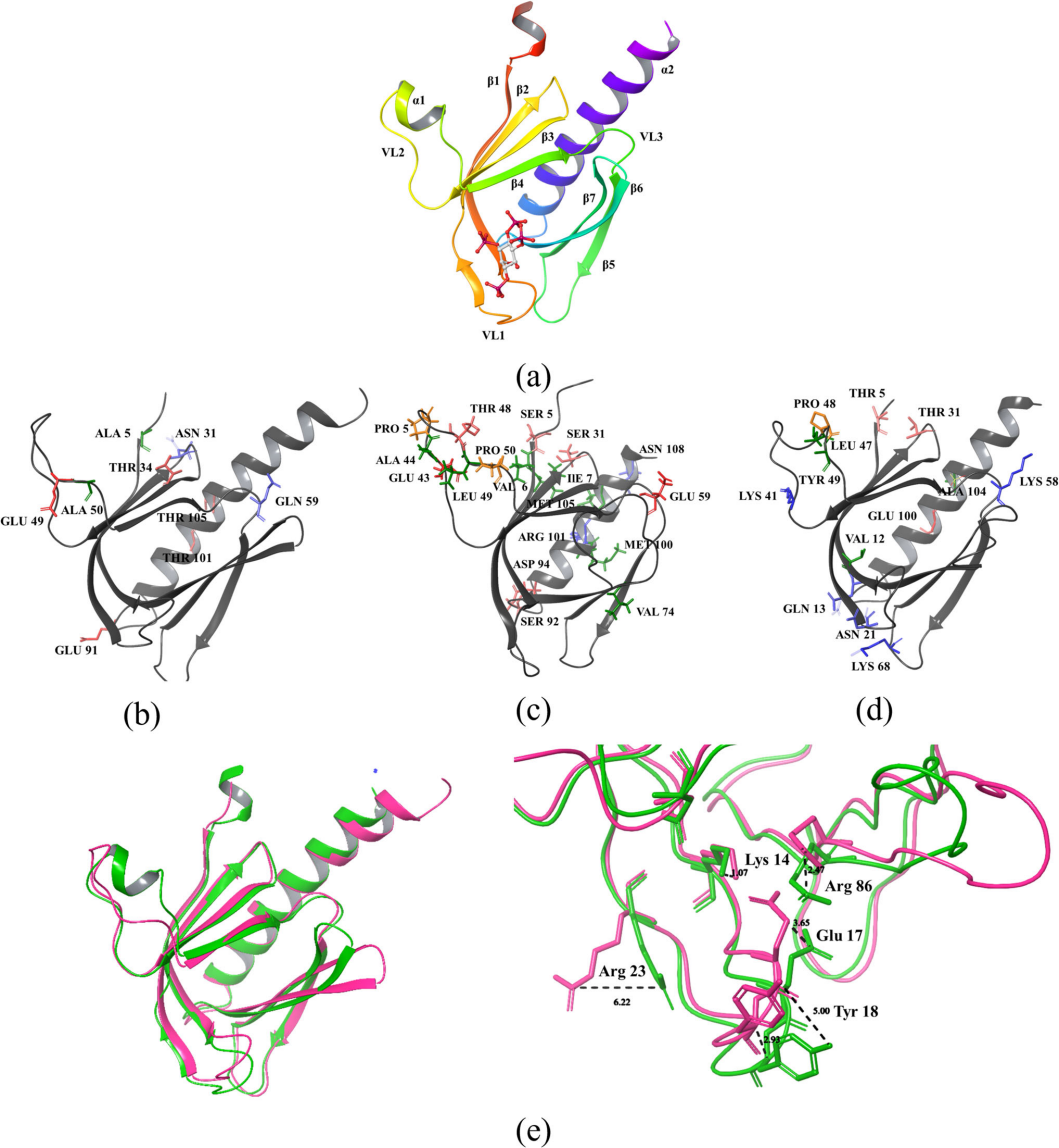
Structural representation of the PH domain. **a** PH domain of AKT1 in active conformation. **b** AKT1. **c** AKT2. **d** AKT3. **e** Comparison of PIP4-bound and Apo conformations of the AKTαPH domain, illustrating structural shifts upon ligand binding

The PH domain is composed of two anti-parallel β-sheets arranged orthogonal comprising seven β-strands which are closed by an α-helix at the C-terminal at one end and by a β-barrel at the other. At the other end of the β-barrel are three loops (VL1–VL3) that exhibit variability in both length and sequence across different PH domains (Fig. 3) [24, 25]. These variable loops form a highly basic pocket into which the head groups of PIP3 and PIP2 can bind with similar affinity but do not interact with phosphatidylinositol-(4,5)-bisphosphate, phosphatidylinositol-3-phosphate or phosphatidylinositol-4-phosphate. The interaction of PIP3 and/or PIP2 does not activate AKT directly but instead brings it into the vicinity of the PDK1. This interaction plays a pivotal role in bringing both enzymes to the plasma membrane in cells stimulated by insulin or growth factors. Additionally, phospholipid binding induces a conformational change in AKT, exposing its activation loop and enabling phosphorylation by PDK1, which is essential for AKT activation [26].

## Structural changes induced by binding of phosphatidylinositol-(1,3,4,5)-tetraphosphate

The Apo AKTαPH domain establishes a complex hydrogen-bonding network involving Lys 14, Glu 17, Asn 53, and Arg 86, along with several water molecules. Upon binding with phosphatidylinositol-(1,3,4,5)-tetraphosphate (PIP4), this hydrogen-bond network is disrupted, leading to significant structural rearrangements. Arg 86 shifts by 2.3 Å toward the 4-phosphate, Lys 14 moves 1.2 Å toward the 3- and 4-phosphates, and Arg 23 migrates 6.2 Å toward the 1- and 3-phosphates. While Asn 53 remains stationary, it directly interacts with the 3- and 4-phosphate groups. Due to its negative charge, Glu17 is repelled by phosphoinositide, prompting a conformational shift in the VL1 region (residues 15–22) and causing Tyr 18 to shift by 2.5 Å in the backbone and 5.0 Å in its side chain to accommodate the 5-phosphate of the PIP4 molecule [26]. This movement also facilitates Arg 86 shift toward the 4-phosphate. The structural differences between the PIP4-bound (1UNQ) and unbound (1UNP) AKTαPH domains are illustrated in Fig. 3e [26].

The D1 phosphate forms limited interactions, binding with Arg 23 and the backbone nitrogen of Ile 19. In contrast, the D3 phosphate interacts extensively with Lys 14, Arg 23, Arg 25, and Asn 53, which may explain AKTαPH inability to bind to PtdIns(4,5)P2, which lacks a D3 phosphate. Similarly, the D4 phosphate engages Lys 14, Asn 53, and Arg 86, highlighting why AKTαPH does not bind to PtdIns(3)P or PtdIns(3,5)P2. Interestingly, the D5 phosphate remains solvent-exposed and interacts exclusively with five ordered water molecules, accounting for AKT’s comparable affinity for both PtdIns(3,4)P2 or PtdIns(3,4,5)P3 [27, 28].

The VL3 loop undergoes a significant conformational adjustment, shifting by up to 7.4 Å toward the phosphoinositide-binding pocket in the ligand-bound structure compared to the apo form. This movement results from the migration of Arg 86 at the base of VL3 toward the 4-phosphate [26]. The hydrophobic residue Trp 80, situated at the VL3 tip, remains solvent-exposed in both apo and complex structures, suggesting a potential interaction site for other AKT sequence regions or AKT-interacting proteins associated with its PH domain [29]. The most notable conformational change involves the VL2 region, where a short acidic α-helix observed in the PIP4-bound complex is absent in the apo structure. Upon PIP4 binding, VL2 undergoes a 7.6 Å shift, enabling the formation of the α-helix. This rearrangement clusters several negatively charged residues (Asp 44, Asp 46, Glu 49, and Glu 40 to a lesser extent) into an acidic patch facing the solvent.

## Comparison with different pH domains

Comparative analyses of AKTαPH with the PH domains of GRP1, BTK, and DAPP1 in complex with PIP4 reveal notable differences in their interaction modes with PIP4. First, the PIP4 molecule within the AKTαPH complex adopts a distinct orientation, rotated approximately 45° in the plane of the inositol ring, and is shifted approximately 4 Å toward the core of the PH domain relative to other PIP4 complexes. This positional shift alters the arrangement of the phosphates and leads to variations in the number and types of interactions. Specifically, the D5 phosphate and D6 hydroxyl group in the AKTαPH complex are fully solvent-exposed, preventing them from forming the hydrogen bonds observed in other structures between the D5 phosphate and the backbone of VL1. Consequently, AKT PH forms fewer interactions with PIP4, which likely accounts for its tenfold lower binding affinity for PIP4 compared to the PH domains of DAPP1, BTK, and GRP1 [30].

**Fig. 4:**
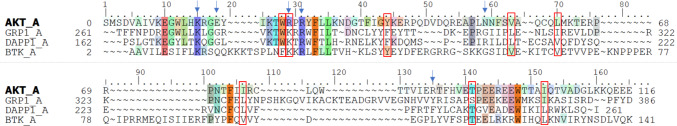
Comparison of PH domains from AKT, GRP1, DAPP1, and BTK, with conserved residues highlighted and homologous residues marked in red around them; arrows denote the residues in PKBαPH involved in interactions with the lipid head group

Another critical difference lies in the conservation of residues involved in binding. While the residues forming interactions with the D4 and D3 phosphates of PIP4 are generally conserved across these PH domains of GRP1 (Tyr 245), DAPP1 (Tyr 195), and BTK (Tyr 39), which contain a conserved tyrosine residue within the binding site that interacts with the D4 phosphate (Fig. 4), but it is absent in AKTαPH. The unique orientation of the head group and the absence of this conserved tyrosine residue could provide valuable insights for designing AKTαPH-specific activators that do not interact with other PH domains [30].

## Kinase domain structure

**Fig. 5:**
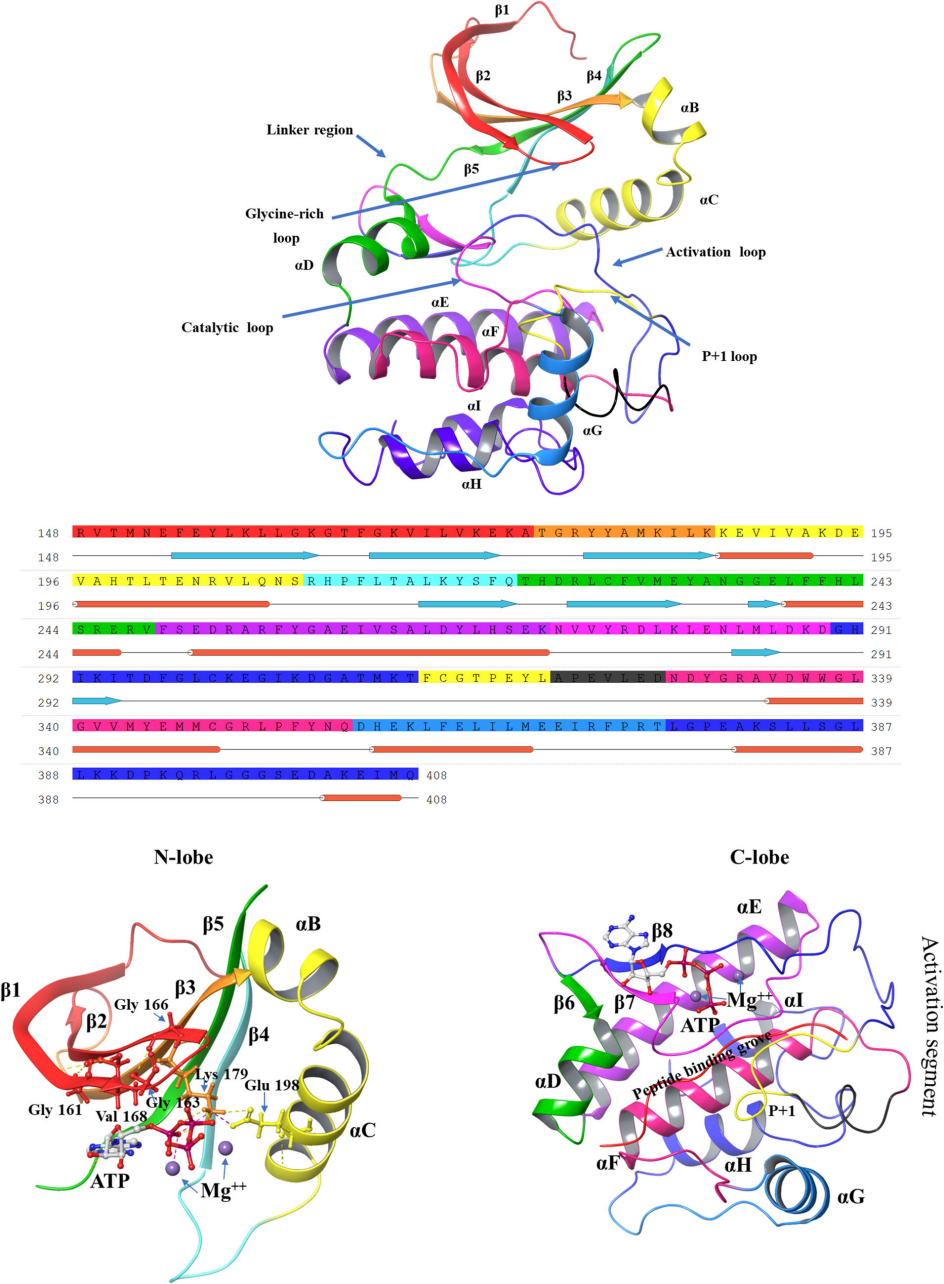
Structure of the kinase domain of AKT1 (C-lobe and N-lobe), with sequence and subdomains color-coded for clarity

**Fig. 6:**
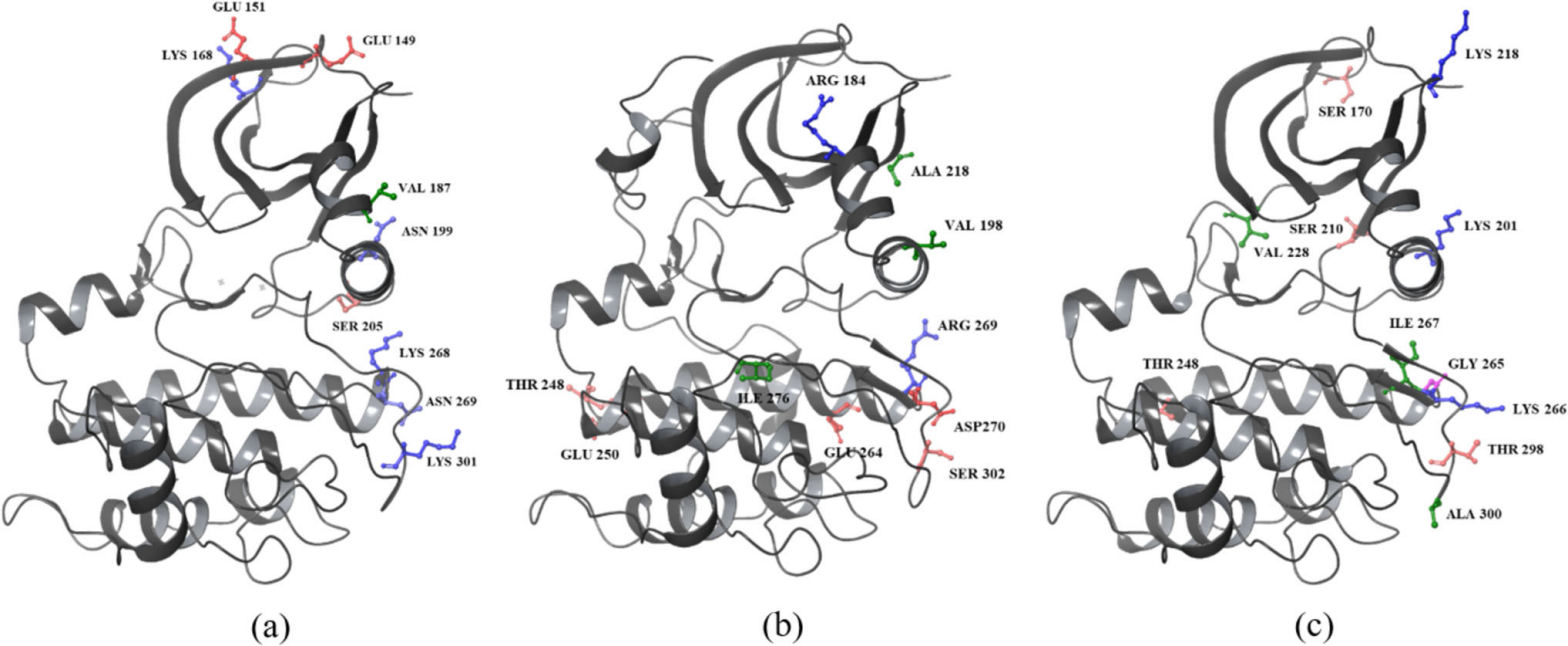
Kinase domain of AKT with unique residues highlighted and colored based on residue types. **a** AKT1. **b** AKT2. **c** AKT3

The kinase domain, a conserved region spanning residues AKT1 (150–408), AKT2 (152–409), and AKT3 (148–405), is structurally divided into two lobes: the smaller N-lobe and the larger C-lobe. The N-lobe consists of four subdomains (I–IV) dominated by a five-stranded β-sheet and a helical subdomain, which includes the C-helix. The larger C-lobe, composed mainly of helices and a β-sheet, contains six subdomains (VI–XI), with subdomain V linking the two lobes [31]. The helical subdomain in the C-lobe forms the stable core of the kinase, serving as a critical tethering surface for protein and peptide substrates [32]. The ATP-binding region of AKT kinases is nearly identical across the isoforms, differing by a single residue: Ala 230 in AKT1, which is conserved as Ala 232 in AKT2 but substituted with Val 228 in AKT3. The structure of the AKT1 kinase domain is described below and illustrated in Fig. 5, while the unique residues specific to this isoform are depicted in Fig. 6.

## N-Lobe

- Subdomain I contains the ATP-binding glycine-rich loop with the GKGTFG motif (Gly 157, Lys 158, Gly 159, Thr 160, Phe 161, Gly 162), connecting β-strands β1 and β2. This highly flexible loop folds over the nucleotide, positioning the γ-phosphate of ATP for catalysis. The loop's tip fully closes only when its backbone is anchored to the γ-phosphate of ATP in a ternary complex. Following the Gly-rich loop, the highly conserved Val 164 makes hydrophobic contact with the ATP base.
- Subdomain II has an important motif AMK (Ala 177, Met 178, Lys 179) within the β3 strand. Lys 179 of this motif links the ATP phosphates to the C-helix.
- Subdomain III is the C-helix, which acts as a "signal integration motif" due to its crucial role in kinase activity and its interactions with various parts of the kinase. In active AKT1, the "DFG-in" conformation allows Glu 179 of Subdomain III to form a highly conserved salt bridge with Lys 179 of Subdomain II and Asp 292 of the DFG motif, a characteristic of the active state. With the C-helix bound to the β-sheet core, the N-lobe exhibits rigid body movement, opening and closing during catalysis. The flexible Gly-rich loop moves with the N-lobe, independent of the kinase's open or closed state. The αC–β4 loop within the N-lobe is a key exception, which remains tightly anchored to the C-lobe.
- Subdomain IV contains a β-strand and contributes to the core structure of the small lobe.

## C-Lobe

- Subdomain V comprises a hydrophobic β-strand in the N-lobe and an α-helix in the C-lobe. The connecting sequence between these two structures contributes residues to both the ATP-binding pocket and the peptide substrate binding site.
- Subdomain VIa contains a long E-helix parallel to the F-helix of Subdomain IX. Subdomain VIb harbors the catalytic loop with the conserved motif YRDLKLEN (Tyr 272, Arg 273, Asp 274, Leu 275, Lys 276, Leu 277, Glu 278, Asn 279). Asp 274 of this motif acts as the catalytic base, accepting the hydrogen from the hydroxyl group being phosphorylated.
- Subdomain VII contains an Mg-binding loop with the DFG motif (Asp 292, Phe 293, Gly 294), which links the two β-strands. The Asp 292 in this motif chelates an Mg2 + ion that bridges the γ and β phosphate of ATP and positions the gamma phosphate for transfer to the substrate.
- Subdomain VIII contains the APE motif (Ala 317, Pro 318, Glu 319). The arginine (Arg 391) in Subdomain XI and the glutamate in the APE motif form a salt bridge that is essential for the formation of the stable kinase core and acts as a crucial step for the movement of the activation loop. The AKT1 has pThr 308 (phosphothreonine) eight residues upstream of the APE motif, which forms an ionic bond with the arginine in the YRDLKLEN motif of the catalytic loop and helps to position it for catalysis. The loop region between Thr 308 and the APE motif is known as the P + 1 loop.
- Subdomain IX contains a hydrophobic F-helix with an invariant aspartate residue (Asp 331). The F-helix serves as a central scaffold for the assembly of the entire protein.
- Subdomain X and Subdomain XI contain G-helix, H-helix, and I-helix forming the kinase core, which binds substrate proteins. Arg 391 present in the H-helix and I-helix loop anchors the GHI domain (Gly 286, His 287, Ile 288) to the activation segment.

## Regulatory spine

The structural alignment of the catalytic cores of PKA and PKB using the Local Spatial Pattern (LSP) approach revealed a highly conserved spatial motif that is essential for kinase activity. This motif comprises four non-consecutive hydrophobic residues, two from the N-lobe (Leu 213 from the β4 strand [33] and Leu 202 from the C-helix), and two from the C-lobe (Phe 293 from the activation loop (DFG motif) and Tyr 272 from the catalytic loop (YRDLKLEN motif)). These residues collectively form a hydrophobic “spine” that structurally connects the two lobes of the kinase.

A key feature of this hydrophobic spine is its dynamic nature. The central elements of the motif the C-helix and the activation loop are highly flexible, allowing the spine to be assembled or disassembled as needed. This structural adaptability plays a crucial role in regulating kinase activity, as it enables transitions between active and inactive states. One of the critical residues of the R-spine, located in the C-lobe, is part of the conserved YRD motif within the catalytic loop. Notably, the backbone of the tyrosine residue (Tyr 272) in this motif is anchored to the F-helix through a conserved aspartate (Asp 331). This aspartate serves as the foundation or “base” of the R-spine, stabilizing the overall kinase structure and ensuring proper catalytic function [34].

## Catalytic spine

Similar to the regulatory (R) spine, this motif consists of residues from both the N-lobe and C-lobe. However, what sets it apart from the R-spine is that it is completed by the adenine ring of ATP, making it an essential structural element for catalytic function [35]. As a result, this motif has been designated the catalytic (C) spine due to its direct involvement in ATP binding and hydrolysis. In the N-lobe, the C-spine consists of two key residues, Val 164 in the β2 strand and Ala 177 from the conserved “AMK” motif within the β3 strand. These residues directly interact with the adenine ring of ATP, facilitating its stable positioning within the catalytic cleft. On the C-lobe side, Met 281, located in the middle of the β7 strand, also docks directly onto the adenine ring. This methionine residue is flanked by two additional hydrophobic residues, Leu 280 and Leu 281 in PKB, further stabilizing the ATP-binding pocket. These β7 hydrophobic residues also rest upon Leu 235, a residue from the D-helix, which is stabilized by hydrophobic interactions with Val 338 and Met 342 from the F-helix. The D-helix plays a crucial role in positioning ATP relative to the rigid hydrophobic core of the C-lobe, ensuring proper catalytic alignment.

## Hydrophobic F-helix

The F-helix is a structural organizer for the kinase core, anchoring the catalytic (C) and regulatory (R) spines. The C-spine connects directly to the hydrophobic C-terminal region of the F-helix, while the R-spine is linked to its N-terminal region. Hydrophobic interactions with the F-helix stabilize several key C-lobe motifs, including the activation segment, catalytic loop, P + 1 loop, and αH-αI loop [34].

In the catalytic loop, Leu 275 and Leu 277 dock directly onto the F-helix, ensuring that the backbone of this loop remains structurally rigid, even in the apo or inactive states. At its C-terminal end lies β7, which contributes to the C-spine [35]. At the N-terminal end is the YRD motif, comprising Tyr 272, Arg 273, and Asp 274. Tyr 272 forms part of the R-spine, Arg 273 interacts with the phosphate in the activation loop of active kinases, and Asp 274 functions either as a weak catalytic base or as a proton trap during substrate phosphorylation. Additionally, the backbone of the YRD motif is anchored to the F-helix via an electrostatic interaction with Asp 331, located at the N-terminal region of the F-helix, reinforcing the hydrophobic network.

## Gatekeeper residue

A "gatekeeper" residue, located between the C- and R-spines, typically near the kinase domain's hinge region on a conserved β5 strand, plays a significant role in protein kinase activation. This residue influences substrate specificity and inhibitor binding by regulating access to the ATP-binding pocket. Acting as a steric barrier, it dictates which molecules can bind based on size and shape. Bulky gatekeeper residues generally favor a closed conformation, while smaller ones allow more open access to the binding pocket. In human kinases, 77% possess bulkier gatekeeper residues like methionine, phenylalanine, and leucine, while 21% of kinases have smaller residues like threonine and valine [36]. In AKT1, Met 227 (Fig. 10d) serves as the gatekeeper, maintaining a relatively constant position regardless of conformational changes.

## DGF motif and C-helix

The DFG motif (Asp 292, Phe 293, Gly 294) within the activation loop is crucial for regulating AKT1 activity. In the active "DFG-in" conformation, Asp 292 binds Mg^2^⁺ ions, and Phe 293 integrates with the R-spine, stabilizing the active kinase domain. Conversely, an inactive "DFG-out" conformation arises from a 180° rotation of Asp 292, causing it and Phe 293 to switch positions (Fig. 10e). This movement displaces Asp 292, 5 Å away from the ATP-binding site, rendering the kinase catalytically inactive. Importantly, this DFG-out state exposes a novel allosteric pocket adjacent to the ATP-binding site, presenting a potential target for allosteric inhibitors [37].

The C-helix, located in the N-lobe of the kinase domain, functions as a "signal integration motif" by dynamically interacting with multiple regions of the kinase. In its active conformation, the C-helix moves inward (αC-in position) (Fig. 10a), facilitating a critical salt bridge between Glu 198 and Lys 179 (β3 strand). This interaction stabilizes the catalytic core and promotes ATP binding. In contrast, when the C-helix adopts an outward orientation (αC-out position), this salt bridge is disrupted, leading to an inactive conformation where ATP binding is impaired.

The transition from an inactive or disordered conformation of the activation loop to an active state (Fig. 10b) is primarily driven by electrostatic interactions, notably the phosphate bridge formation with Arg 273. Upon activation, Asp 292 is optimally positioned for catalysis, while Phe 293 aligns with Tyr 272 (YRD motif) and Leu 202 (C-helix). This alignment links the N- and C-lobes, coordinating their function and priming the enzyme for catalysis. The stabilization of Phe 293 within the R-spine reinforces the active conformation and facilitates entropy-driven communication between the C- and N-lobes, ensuring efficient signal transduction and catalytic activity [38]. The conformations of all crystal structures of the kinase domain in AKT1 have been analyzed and are summarized in Table 2**.**

**Table 2:** Conformational states of the AKT1 kinase domain

PDB entry	Title	Conformation state	Resolution (Å)	Chain	Positions
3CQU	Crystal structure of AKT-1 complexed with substrate peptide and inhibitor	Active	2.20	A	144–480
3CQW	Crystal structure of AKT-1 complexed with substrate peptide and inhibitor	Active	2.00	A	144–480
3MV5	Crystal structure of AKT-1-inhibitor complexes	Active	2.47	A	144–480
3MVH	Crystal structure of AKT-1-inhibitor complexes	Active	2.01	A	144–480
3O96	Crystal structure of Human AKT1 with an Allosteric Inhibitor	Inactive, missing C-Helix structure	2.70	A	2–443
3OCB	AKT1 kinase domain with pyrrolopyrimidine inhibitor	Active	2.70	A/B	144–480
3OW4	Discovery of dihydrothieno- and dihydrofuropyrimidines as potent pan AKT inhibitors	Active	2.60	A/B	144–480
3QKK	Spirochromane AKT inhibitors	Active	2.30	A	144–480
3QKL	Spirochromane AKT inhibitors	Active	1.90	A	144–480
3QKM	Spirocyclic sulfonamides as AKT inhibitors	Active	2.20	A	144–480
4EJN	Crystal structure of autoinhibited form of AKT1 in complex with *N*-(4-(5-(3-acetamidophenyl)-2-(2-aminopyridin-3-yl)-3H-imidazo[4,5-b]pyridin-3-yl)benzyl)-3-fluorobenzamide	Inactive, missing C-Helix structure	2.19	A	2–446
4EKK	AKT1 with AMP-PNP	Active	2.80	A/B	144–480
4EKL	AKT1 with GDC0068	Active	2.00	A	144–480
4GV1	PKB alpha in complex with AZD5363	Active	1.49	A	144–480
5KCV	Crystal structure of allosteric inhibitor, ARQ 092, in complex with autoinhibited form of AKT1	Inactive, missing C-Helix structure	2.70	A	2–446
6BUU	Crystal structure of AKT1 (aa 144–480) with a bisubstrate	Active	2.40	A/B	144–480
6CCY	Crystal structure of AKT1 in complex with a selective inhibitor	Active	2.18	A	144–466
6HHF	Crystal structure of AKT1 in complex with covalent-allosteric AKT inhibitor Borussertib	Inactive, missing C-Helix structure	2.90	A	2–446
6HHG	Crystal structure of AKT1 in complex with covalent-allosteric AKT inhibitor 27	Inactive, missing C-Helix structure	2.30	A	2–446
6HHH	Crystal Structure of AKT1 in Complex with Covalent-allosteric AKT inhibitor 31	Inactive, missing C-Helix structure	2.70	A	2–446
6HHI	Crystal structure of AKT1 in complex with covalent-allosteric AKT inhibitor 30b	Inactive, missing C-Helix structure	2.70	A	2–446
6HHJ	Crystal structure of AKT1 in complex with covalent-allosteric AKT inhibitor 24b	Inactive, missing C-Helix structure	2.30	A	2–446
6NPZ	Crystal structure of AKT1 (aa 123–480) kinase with a bisubstrate	Active	2.12	A/B	123–480
6S9W	Crystal structure of AKT1 in complex with covalent-allosteric AKT inhibitor 16a	Inactive	2.30	A	2–446
6S9X	Crystal structure of AKT1 in complex with covalent-allosteric AKT inhibitor 15c	Inactive, missing C-Helix structure	2.60	A	2–446
7APJ	Structure of autoinhibited AKT1 reveals mechanism of PIP3-mediated activation	Missing DGF motif and C-helix	2.05	A	5–434
7NH4	Co-crystal structure of AKT1 in complex with covalent-allosteric AKT inhibitor 3	Inactive, missing C-Helix structure	2.30	A	2–446
7NH5	Co-crystal structure of AKT1 in complex with covalent-Allosteric AKT Inhibitor 6	Inactive, missing C-Helix structure	1.90	A	2–446
8UVY	Structure of AKT1(E17K) with compound 3	Inactive, missing C-Helix structure	2.11	A	2–446
8UW2	Structure of AKT1(E17K) with compound 3 (zinc-free)	Inactive, missing C-Helix structure	2.20	A	2–446
8UW7	Structure of AKT1(WT) with compound 3	Inactive, missing C-Helix structure	1.97	A	2–446
8UW9	Structure of AKT1(E17K) with compound 4	Inactive, missing C-Helix structure	1.90	A	2–446

## C-terminal tail

The AGC kinase C-terminal domain present at the C-terminal of AKT1 consists of 71 amino acids (409–480) and possesses the FPQFSY hydrophobic motif (Phe 469, Pro 470, Gln 471, Phe 472, Ser 473, Tyr 474) that is characteristic of the AGC family of protein kinases. This motif is crucial for AKT full activation, while phosphorylation of the activation loop (Thr 308) is a primary activation step mediated by PDK1, subsequent phosphorylation of the hydrophobic motif serine residue (Ser 473) is essential for maximal kinase activity [39].

The activation of pSer473-AKT is driven by an intramolecular interaction between the PH-kinase domain linker and the C-tail, which relieves AKT1 autoinhibition [40]. Notably, PDK1 cannot phosphorylate wild-type AKT under conditions where it efficiently phosphorylates a mutant form of AKT lacking its PH domain, highlighting the importance of this domain in AKT activation [41, 42]. Although monophosphorylated pT308 AKT retains only 10% of the reactive ability compared to the fully activated diphosphorylated state, phosphorylation of Thr 308 is crucial for AKT catalysis. Phosphorylated Thr 308 can also resist dephosphorylation by interacting with His 194 in the αC helix and Arg 273 (YRD motif) in the catalytic loop [43].

Structural studies indicate that phosphorylation at Ser 473 facilitates interaction with a basic patch in the PH-kinase domain linker, particularly involving the side chain of Arg 144, and with the kinase N-lobe via Gln 218. This interaction aids in displacing the PH domain from the kinase domain, thereby enhancing catalytic activity [40]. Additionally, phosphorylation at Ser 477 and Thr 479 in AKT1, mediated by Cdk2/cyclin A, appears to relieve autoinhibition through a distinct mechanism, possibly involving interactions with the activation loop and/or the PH domain [44]. Furthermore, Ser 473 in AKT1 can be phosphorylated by DNA-PK in the nucleus as part of the DNA damage response activation [45].

## AlphaFold structure

AlphaFold, developed by DeepMind, is a revolutionary artificial intelligence system designed to predict the three-dimensional structures of proteins with remarkable accuracy. Utilizing deep learning techniques, AlphaFold solves the protein folding problem by predicting atomic-level protein structures based solely on their amino acid sequences. It has significantly advanced structural biology, enabling insights into protein functions and interactions that were previously difficult to achieve through experimental methods alone [46].

## Predicted local distance difference test

The predicted local distance difference test (pLDDT) is a crucial metric used to evaluate the confidence of protein structure predictions generated by advanced computational methods like AlphaFold. As a per-residue measure, pLDDT provides insights into the local accuracy of predicted structures, offering scores on a scale from 0 to 100. Higher scores indicate greater confidence, suggesting that the predicted distances between a residue and its neighbors are likely to closely match the true distances as would be observed in experimental structures. Conversely, low pLDDT scores indicate uncertainty or potential inaccuracies in the local structural prediction. pLDDT is grounded in the principles of the local distance difference test Cα (lDDT-Cα), which assesses the correctness of local distances without requiring superposition of the structures being compared [47]. This reliance on internal coordinates comparing inter-atomic distances within one structure to those in another makes pLDDT a robust and objective tool for evaluating structure predictions, free from the need for manual intervention.

**Fig. 7:**
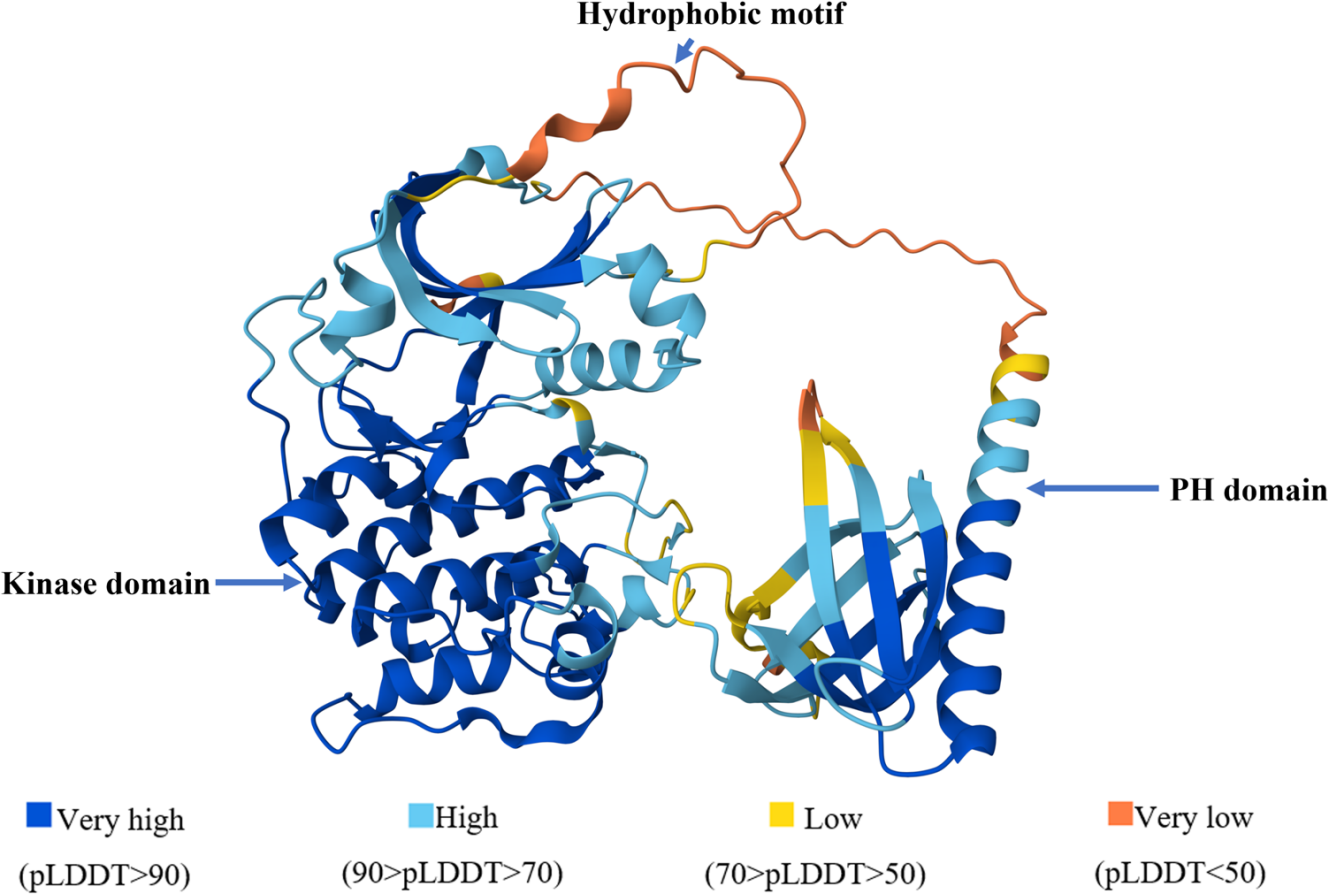
Completed domain structure of AKT1 predicted by AlphaFold, colored based on per-residue model confidence scores (pLDDT)

The interpretation of pLDDT scores provides valuable guidance for different applications. Regions with pLDDT scores above 90 are expected to be modeled with high accuracy, making them suitable for detailed applications, such as characterizing binding sites. Scores between 70 and 90 indicate well-modeled regions, generally reflecting a good backbone prediction. In contrast, regions scoring between 50 and 70 suggest low confidence and show potential skepticism about the reliability of the prediction in these areas [48]. AlphaFold2 assigns low-confidence scores to protein regions for two primary reasons. First, the region may naturally lack a well-defined structure due to being highly flexible or intrinsically disordered. Second, the region might possess a predictable structure, but AlphaFold2 may lack sufficient data to confidently predict it. In either case, these regions typically receive a pLDDT score below 50. Linker regions, in particular, tend to exhibit natural variability, reduced structural organization, and greater flexibility. There is no way to predict specific structures for such naturally unstructured regions, so AlphaFold2 assigns its predictions a low confidence. Well-defined structure or there may be insufficient information to predict the structure with confidence. Both scenarios usually result in pLDDT scores below 50. Naturally variable regions, such as linkers, are often less structured and more flexible, making them challenging to predict with high confidence. As a result, AlphaFold often assigns lower pLDDT scores to these regions, reflecting the inherent difficulty in predicting specific structures for naturally unstructured areas. Understanding these nuances of pLDDT scoring is essential for researchers, as it provides a clear framework for interpreting the confidence and reliability of computationally predicted protein structures. The Per-residue model confidence score (pLDDT) of AlphaFold’s structure of AKT1 for each residue is tabulated in Table 3 and illustrated in Fig. 7.

**Table 3:**
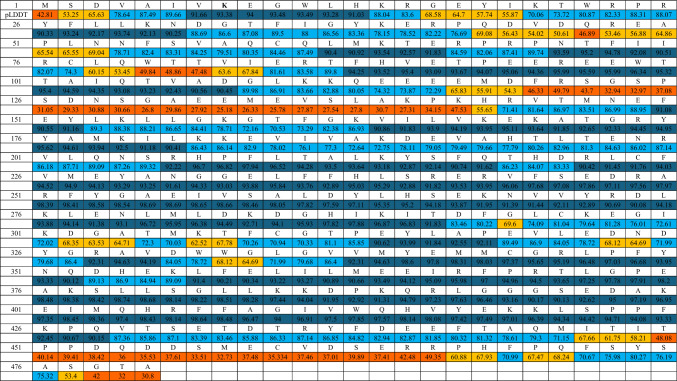
Per-residue model confidence scores (pLDDT) for the AlphaFold-predicted structure of AKT1, highlighting regions of varying structural confidence

## Predicted local distance difference test for AKT1 (AF-P31749)

The PH domain of AKT1 exhibits several regions with low pLDDT values, indicating flexibility and potential conformational changes. Residues 1, 47, 80–82 have particularly very low pLDDT, suggesting high flexibility. Other regions with low pLDDT include 2–3, 16–19, 43–46, 48–53, 78–79, 83–84. The linker region also shows regions with very low pLDDT (120–142). Specific residues within the PH domain have been implicated in conformational changes and interactions. Glu 17 and Tyr 18 are involved in conformational changes induced by PtdIns(1,3,4,5)P4 binding. Asp 44, Asp 46, and Glu 49 form an acidic patch. The Trp 80 is a hydrophobic residue located at the tip of VL3 which is completely solvent-exposed. Notably, most residues showing low pLDDT in the PH domain are found at the interface with the kinase domain, suggesting their involvement in interdomain interactions.

The kinase domain of AKT1 exhibits several regions having low pLDDT values. Residues 143, 294, 302–304, 307–308, 323–324, and 333–334 fall within this category. These regions play crucial roles in AKT1’s catalytic activity and structural integrity. Gly 294 is a key residue within the DGF motif, which forms a catalytic triad in the active conformation. The DGF motif is highly conserved among protein kinases and is essential for ATP binding and catalysis. Residues 302–304 and 307–308 are part of the activation loop, a flexible region that undergoes conformational changes upon phosphorylation. Phosphorylation of Thr 308 within this loop is a critical step in AKT1 activation. Residues 323–324 and 333–334 are located within the F-helix, a structural element that serves as a central scaffold for the assembly of the entire kinase domain. The F-helix contributes to the overall stability and proper folding of the kinase domain, ensuring its catalytic function.

Several regions within the hydrophobic motif exhibit low pLDDT values, indicating flexibility and potential conformational changes. Residues 450–466 and 478–480 have particularly very low pLDDT. Other regions with low pLDDT include 447–449, 467, 468, 470, 471, and 477. The flexibility of the hydrophobic motif is likely essential for its role in AKT1 activation. It allows for the binding of regulatory proteins and the adoption of different conformations that are necessary for full kinase activity. Mutations within the hydrophobic motif that disrupt its flexibility, or binding properties can impair AKT1 activation and lead to pathological consequences.

## Predicted aligned error for AKT1 (AF-P31749) AlphaFold structure

Predicted aligned error (PAE) provides a visual representation of the confidence in the relative positions of residues within a predicted protein structure. The heatmap illustrates the Predicted Aligned Error (PAE) for a protein structure prediction, offering insights into the expected positional error between residue pairs. The *x*-axis represents scored residue positions, while the *y*-axis represents aligned residue positions. The color scale at the bottom indicates the expected positional error in Angstroms, with darker shades of green denoting lower errors and hence more reliable predictions, and lighter shades signifying higher errors and less reliable predictions [49].

A diagonal line running from the bottom left to the top right indicates the same residue aligned to itself, typically showing lower errors due to higher prediction confidence. The dark green diagonal line running from the top-left (PH domain) to the bottom-right (kinase domain) of the heatmap indicates areas where the predicted structure has high confidence. These are typically regions where residues are close to each other in sequence and are predicted to have accurate relative positioning. This suggests that these parts of the protein likely form stable, well-defined structures, such as alpha-helices or beta-sheets.

**Fig. 8:**
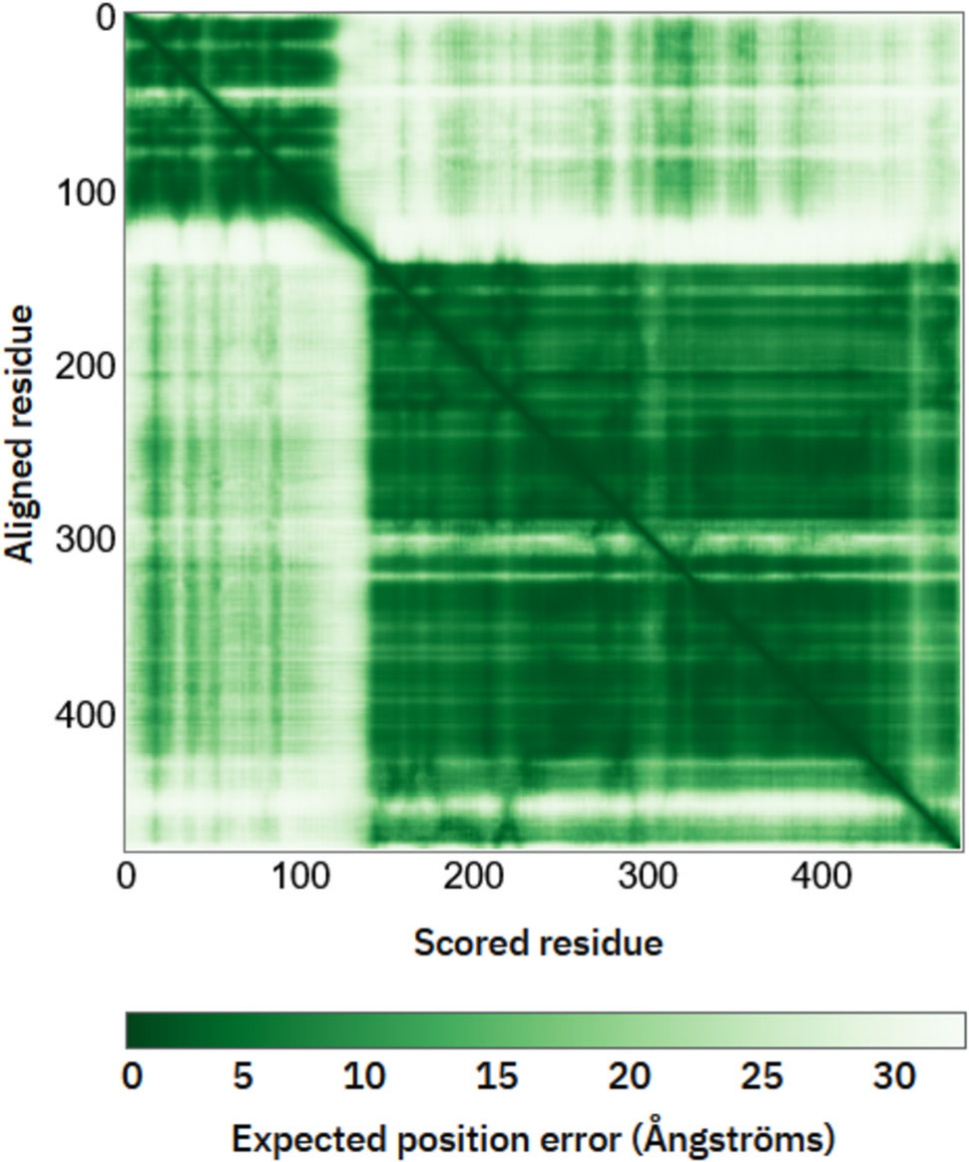
Predicted Aligned Error (PAE) heatmap for the AlphaFold-predicted structure of AKT1, illustrating relative confidence in residue positioning

The lighter areas, particularly in the upper left and lower right corners of the heatmap, indicate regions of lower confidence in the relative positioning of the residues. These areas represent a flexible loop between the PH domain and kinase domain, domain interfaces between PH and kinase domain, and regions with potential conformational variability like hydrophobic motif. The higher PAE values here suggest that the model is less certain about how these residues are positioned relative to each other and the domain movements or interactions that are not well resolved in the model (Fig. 8).

## Sequential mechanism and features of AKT1 activation

**Initial state:** AKT1 exists in its inactive state, with the conserved DFG motif in the kinase domain adopting the DFG-out conformation. This inactive state is stabilized through interactions between the PH domain and the kinase domain in the PH-in conformation, which positions the DFG motif to block the ATP-binding site [50].

**Membrane translocation:** AKT1 translocates to the plasma membrane upon activation signals such as PI3K activation. This relocation is facilitated by the binding of the PH domain to phosphatidylinositol lipids (PIP3 or PIP2), which are enriched in the membrane. The interaction induces conformational changes in AKT1, preparing it for activation.

**PH domain relocation:** The PH domain undergoes a positional shift, moving away from the kinase domain to adopt a PH-out conformation. This repositioning alleviates steric hindrance and permits the reorganization of the kinase domain, particularly the activation loop and catalytic cleft [50, 51]. The DFG motif transitions from the DFG-out to the DFG-in conformation, positioning the phenylalanine residue outside the ATP-binding site and allowing the aspartic acid residue to coordinate ATP-associated metal ions. ASP 293, Lys 179, and Glu 198 salt formation takes place.

**ATP binding:** ATP binds to the ATP-binding cleft in the kinase domain, inducing a conformational change where the N- and C-lobes of the kinase domain shift from open to closed conformation. This structural adjustment aligns catalytic residues and primes AKT1 for efficient catalysis.

**Hydrogen bond formation:** The hydroxyl side chain of Thr 160, located in the G-loop, forms a critical hydrogen bond with the β-phosphate moiety of ATP exposing the γ-phosphate. These interactions stabilize ATP binding and position the γ-phosphate for effective transfer to substrates.

**Fig. 9:**
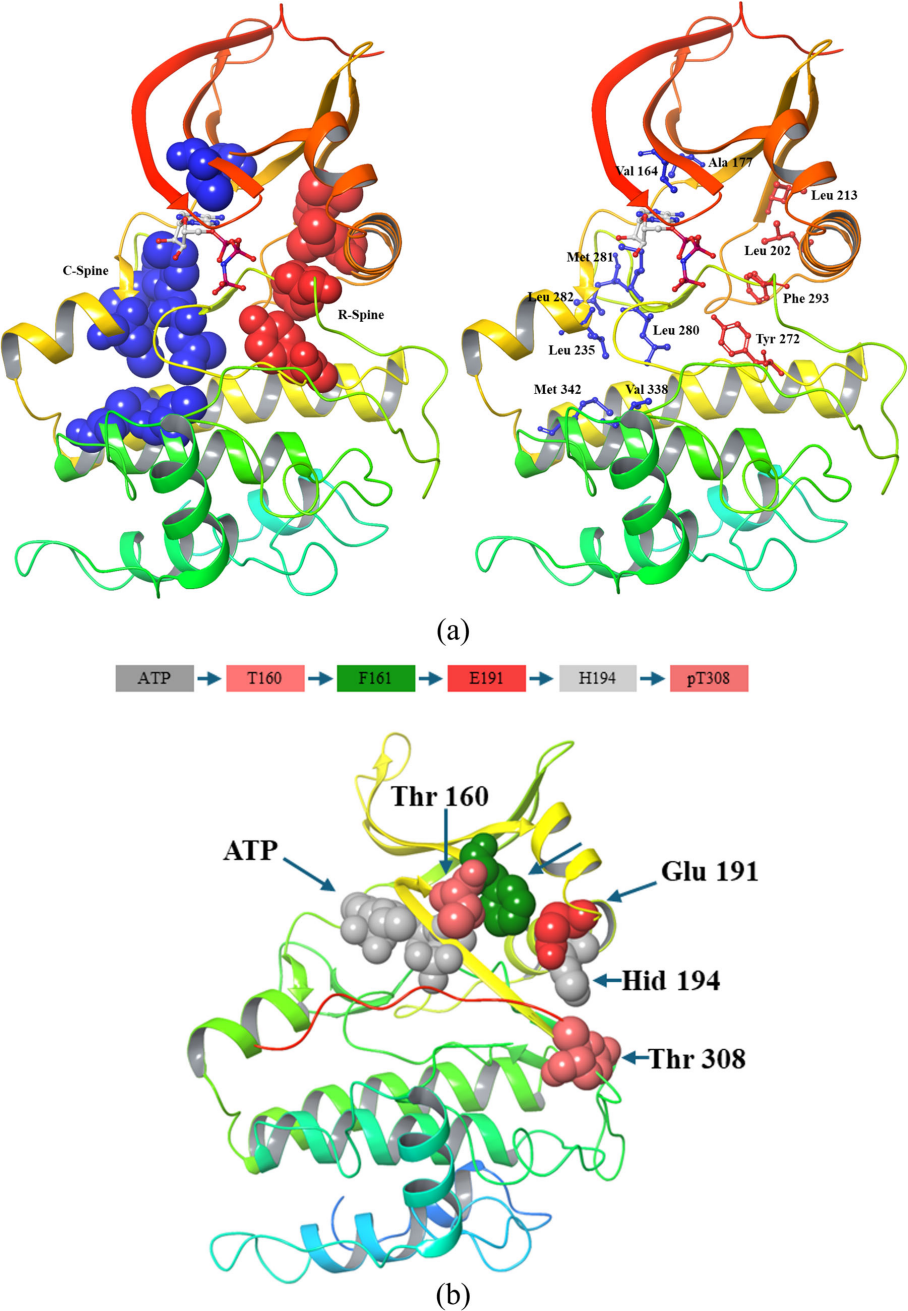
Kinase domain structural features **a** R-Spine and C-Spine formed in active conformation in Kinase domain and AlphaFold-predicted AKT1 structure of AKT1; **b** Allosteric pathway by which phosphorylation of Thr 308 is protected

**Activation loop phosphorylation:** Phosphorylation of Thr 308 in the activation loop occurs, catalyzed by PDK1. The phosphorylated Thr 308 (pT308) is encapsulated by three positively charged residues His 194, Arg 273, and Lys 297 within the catalytic cleft in the closed conformation of the kinase domain. These residues shield pT308 from dephosphorylation and stabilize the active conformation of AKT1. ATP occupancy in the ATP-binding cleft allosterically protects pThr 308 (ATP → Thr 160 → Phe 161 → Glu 191 → His 194 → pThr 308) from dephosphorylation (Fig. 9b) [52]. Residues involved in the pathway are scattered on different parts of AKT1: Thr 160 and Phe 161 are located in the G-loop, Glu 191 at the junction of the αB and αC helices, and His 194 in the αC helix. The side-chain carboxyl group of Glu 191 forms a hydrogen bond with the imidazole ring of His 194, which donates an additional hydrogen bond to the phosphate group of pT308, ensuring its stability. Conversely, the binding of ADP or the absence of nucleotide binding results in the loss of this protection and a transition to the inactive state [53].

**Hydrophobic motif phosphorylation:** Phosphorylation of Ser 473 in the hydrophobic motif is mediated by mTORC2. This modification further stabilizes the active conformation by enhancing interactions between the hydrophobic motif and the kinase domain. pSer 473 concurrently interacts with a PH-kinase domain linker basic patch, especially the side chain of Arg 144, and the kinase N-lobe via Gln 218 helping to displace the PH domain from the kinase domain thereby stimulating catalytic activity [40].

**Phosphate transfer reaction:** The γ-phosphate group of ATP is transferred to the substrate during the phosphorylation reaction. This process is facilitated by the aligned and stabilized catalytic site, completing the enzymatic activity of AKT1.

## Essential features for the AKT1 active state

**Fig. 10:**
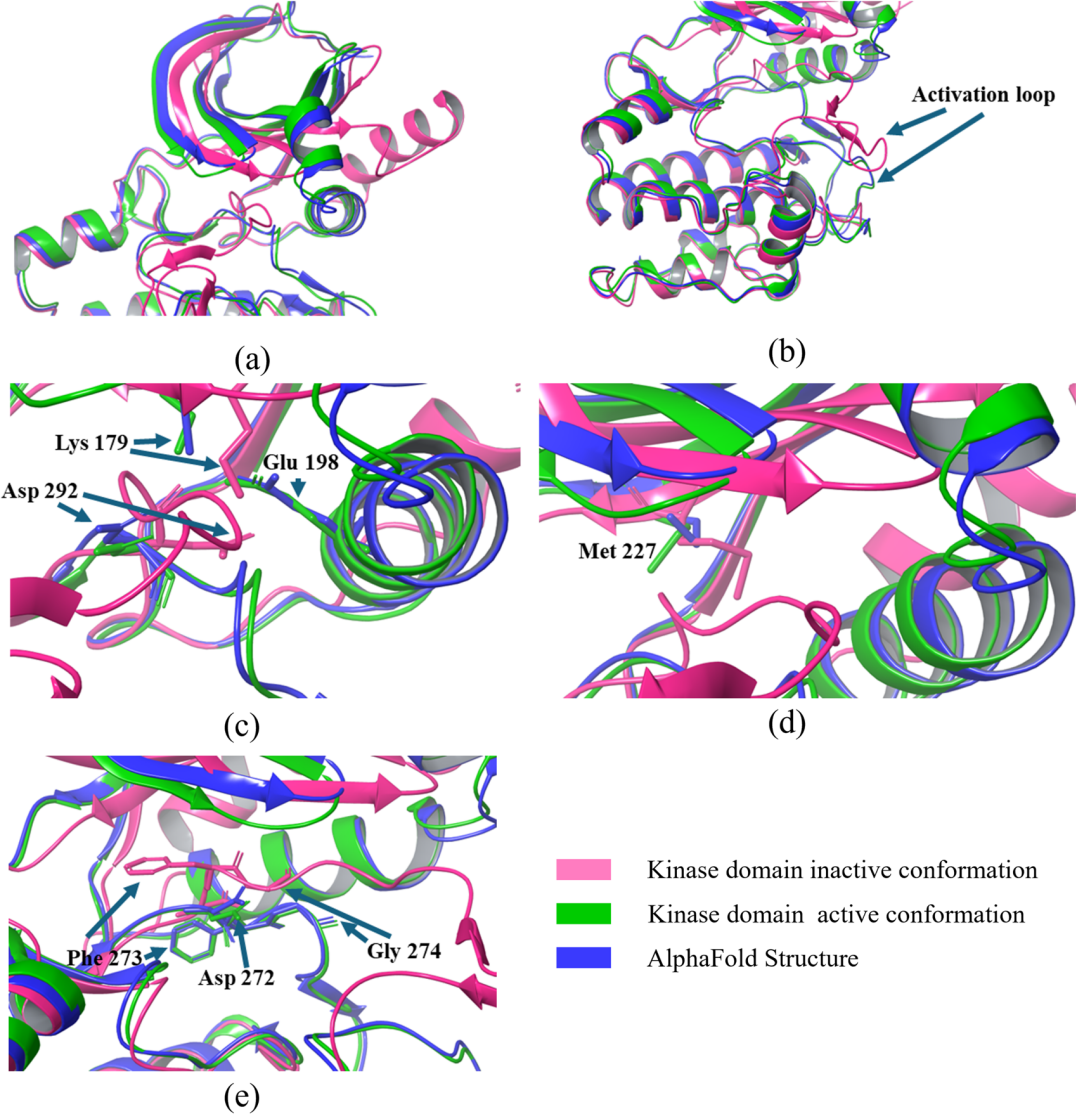
Comparison of the kinase domain of AKT in an active, inactive conformation and Alphafold’s predicted structure. **a** Position of the αC-helix, showing an inward orientation in the active state and an outward shift in the inactive state; **b** Position of Activation loop; **c** comparison of triad formation between Glu 198, Lys 179, and Asp 292; **d** Positioning of gatekeeper residue (Met 227); **e** DFG-motif conformation, illustrating the DFG-in state in the active form and DFG-out conformation in the inactive form.;

The active conformation of AKT1 relies on several critical features, many of which are absent in the structure predicted by AlphaFold. One major limitation is the absence of ATP, which is essential for aligning catalytic residues and stabilizing the active state. Although the salt bridge involving Asp 292, Lys 179, and Glu 198 is preserved (Fig. 10c), other key elements are missing. Substrate peptide which is essential for stabilizing active conformation is also missing. Phosphorylation of Thr 308 and Ser 473, crucial for stabilizing the activation loop and the hydrophobic motif, respectively, is absent. Additionally, the interactions of phosphorylated pThr 308 with His 194 in the αC helix and Arg 273 (part of the YRD motif) are absent. The doubly protonated His 194, which performs critical functions in the active state, is also missing. Specifically, the NεH of His 194 protects pThr 308, while the NδH interacts with Glu 191 to maintain the structural stability of the helix. Key allosteric interactions vital for AKT1 activation are not observed in the AlphaFold structure, including those involving Thr 160 and Phe 161 in the G-loop, Glu 191 at the αB/αC helix junction, and His 194 in the αC helix.

Furthermore, magnesium ions are absent, which are crucial in coordinating ATP binding and catalysis. The shielding interaction between His 194, Arg 273, and Lys 297, which protects pThr 308 from dephosphorylation, is also missing. Another notable omission is the absence of water molecules in the ATP-binding pocket, which are essential for facilitating the transition to a catalytically ready state. However, the AlphaFold structure does exhibit the DFG-in conformation of the kinase domain, a hallmark of the active state in which the phenylalanine residue shifts out of the ATP-binding site, and the aspartic acid residue coordinates ATP-associated metal ions. Additionally, the R-spine residues (Leu 202, Phe 293, Leu 213, Tyr 272) and S-spine residues (Val 164, Ala 177, Met 281, Leu 282, Leu 280, Leu 235, Val 338, Met 342) align as expected in the active conformation.

**Fig. 11:**
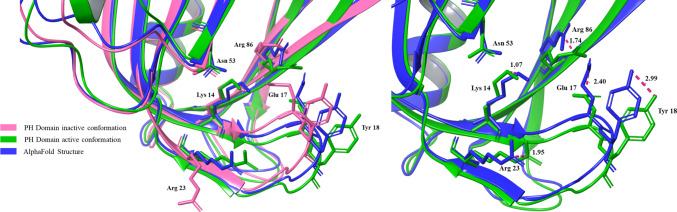
Comparison of the PH domain in active, inactive, and AlphaFold-predicted structures, focusing on conformational and positional deviations

The PH domain also exhibits deviations from the active conformation. It is not positioned in the PH-out conformation, a state that facilitates effective ATP binding and proper alignment of the kinase domain for catalysis. Structural differences include the absence of the small helix at VL2 and a shift in VL1, although shifts in β6 and β7 are not observed. Additionally, important residues such as Asn 53, Lys 14, Arg 23, Arg 86, Glu 17, and Tyr 18 show slight positional shifts, as illustrated in the accompanying Fig. 11. Lastly, the hydrophobic motif lacks key interactions involving pSer 473 with Arg 144 and Gln 218, which are essential for its stabilization. These deviations collectively highlight significant gaps in the AlphaFold structure compared to the active state of AKT1.

## Limitations

Despite significant advancements in understanding AKT1 structure and activation mechanisms, several challenges remain. The absence of a full-length crystal structure of AKT in its active conformation due to flexibility of the PH domain when binds to PIP3 or PIP2 and takes PH-out conformation impedes precise structural analysis and restricts the development of AKT activators. Although available crystal structure in which AKT is bound to phosphoaminophosphonic acid-adenylate ester (AMP-PNP) [54] and PH domain bound to PIP4 [26], experimental studies and molecular dynamics simulations [53] provide valuable insights into AKT1 conformational dynamics, but modeling full AKT structure experimental validation remains a challenge due to the transient nature of conformational changes and phosphorylation events. Additionally, most structural studies focus on AKT1, with limited insights into the other isoforms, AKT2 and AKT3, which play distinct physiological roles. This gap in structural data hinders the development of isoform-specific therapeutics. While several small-molecule AKT activators have been identified, issues such as poor specificity and limited bioavailability, as seen with compounds like SC79, present significant obstacles to their clinical application. The AlphaFold-predicted AKT1 structure [55], while informative, lacks key elements of the active state. These omissions limit the accuracy of computational models in representing AKT1 activation and its interactions with regulatory molecules.

## Future prospects for the modeling of AKT structure

To overcome these limitations, future research should focus on structural determination of PH domain of AKT1 bound to know activators like SC79 [11], chlorogenic acid [18], puerarin [19], quercetin-3-glucuronide [20], baicalin [21], kaempferol-3-glucuronide [20], ferulic-4-*O*-glucuronide [22], and Caffeic-4-*O*-glucuronide. Advanced techniques like cryo-electron microscopy (Cryo-EM) and NMR spectroscopy should be employed to obtain high-resolution structures, providing deeper insights into the activation mechanisms of these molecules. These structural insights will not only clarify the molecular basis of AKT activation but also provide a foundation for the rational design of novel AKT activators.

The AlphaFold-predicted AKT1 structure can be refined by incorporating key missing elements, including ATP, magnesium ion coordination, phosphatidylinositol-(1,3,4,5)-tetraphosphate, phosphorylation at Ser 473 and Thr 308, and a substrate peptide. These modifications will allow for computational modeling using molecular dynamics (MD) simulations, followed by experimental validation to improve structural accuracy. Additionally, homology modeling can be performed by merging the kinase domain (PDB ID: 4EKK) and PH domain (PDB ID: 1UNQ), using them as templates to construct a full-length AKT1 structure in its active conformation. Subsequent MD simulations and induced fit docking will further refine the active-state model, facilitating drug design efforts.

A major challenge in computational validation is the lack of structurally similar, experimentally validated inactive ligands for AKT1. Currently, only a few inactive ligands related to SC79 have been reported, which is insufficient for rigorous validation. To address this, future studies should focus on identifying structurally similar compounds that fail to activate AKT1 by binding to the PH domain. Expanding the dataset of both active and inactive ligands will improve the reliability of in silico screening models.

By integrating high-resolution structural biology, computational modeling, and AI-driven drug discovery, future research can identify new AKT activators. As our understanding of AKT activation deepens, we move closer to developing selective activators that could open new avenues for treatments of cancer, diabetes, and neurodegenerative disorders.

## Conclusion

This study provides comprehensive insights into the structural and activation mechanisms of AKT1, a pivotal serine/threonine kinase in cellular signaling pathways. The findings elucidate critical features required for AKT1’s active conformation, including the DFG-in state, PH-out domain movement, and dual phosphorylation at Thr 308 and Ser 473, which collectively stabilize the kinase for efficient substrate phosphorylation. Notably, the limitations of AlphaFold’s AKT1 structure, such as the absence of phosphorylation, ATP binding, manganese coordination, and allosteric interactions (ATP → Thr 160 → Phe 161 → Glu 191 → His 194 → pThr 308), highlight the challenges in computational modeling of dynamic protein states. By bridging structural insights with activation mechanisms, this study highlights the importance of these elements in AKT1-mediated signaling. The identified insights will serve as a roadmap for refining the AlphaFolds AKT structure in active conformation by using computational tools like molecular dynamic simulation after making the necessary changes highlighted in the review and developing targeted therapeutics aimed at activation of AKT1 activity. These findings hold relevance for addressing diseases associated with AKT pathway dysregulation, including cancer, diabetes, and neurodegenerative disorders. Future studies integrating experimental and computational approaches are essential to advancing our understanding and leveraging AKT1 as a therapeutic target.

## Data Availability

No datasets were generated or analyzed during the current study.
